# Identification, heterologous expression and characterization of a new unspecific peroxygenase from *Marasmius fiardii* PR-910

**DOI:** 10.1186/s40643-024-00751-x

**Published:** 2024-03-27

**Authors:** Xin Fu, Kexin Lin, Xiaodong Zhang, Zhiyong Guo, Lixin Kang, Aitao Li

**Affiliations:** State Key Laboratory of Biocatalysis and Enzyme Engineering, Hubei Key Laboratory of Industrial Biotechnology, School of Life Sciences, Hubei University, #368 Youyi Road, Wuhan, 430062 People’s Republic of China

**Keywords:** Unspecific peroxygenase, *Marasmius fiardii*, Heterologous expression, Characterization, Semi-preparative

## Abstract

Unspecific peroxygenases (UPOs) are glycosylated enzymes that provide an efficient method for oxyfunctionalizing a variety of substrates using only hydrogen peroxide (H_2_O_2_) as the oxygen donor. However, their poor heterologous expression has hindered their practical application. Here, a novel UPO from *Marasmius fiardii* PR910 (*Mfi*UPO) was identified and heterologously expressed in *Pichia pastoris*. By employing a two-copy expression cassette, the protein titer reached 1.18 g L^−1^ in a 5 L bioreactor, marking the highest record. The glycoprotein r*Mfi*UPO exhibited a smeared band in the 40 to 55 kDa range and demonstrated hydroxylation, epoxidation and alcohol oxidation. Moreover, the peroxidative activity was enhanced by 150% after exposure to 50% (v/v) acetone for 40 h. A semi-preparative production of 4-OH-β-ionone on a 100 mL scale resulted in a 54.2% isolated yield with 95% purity. With its high expression level, r*Mfi*UPO is a promising candidate as an excellent parental template for enhancing desirable traits such as increased stability and selectivity through directed evolution, thereby meeting the necessary criteria for practical application.

**Graphical Abstract:**

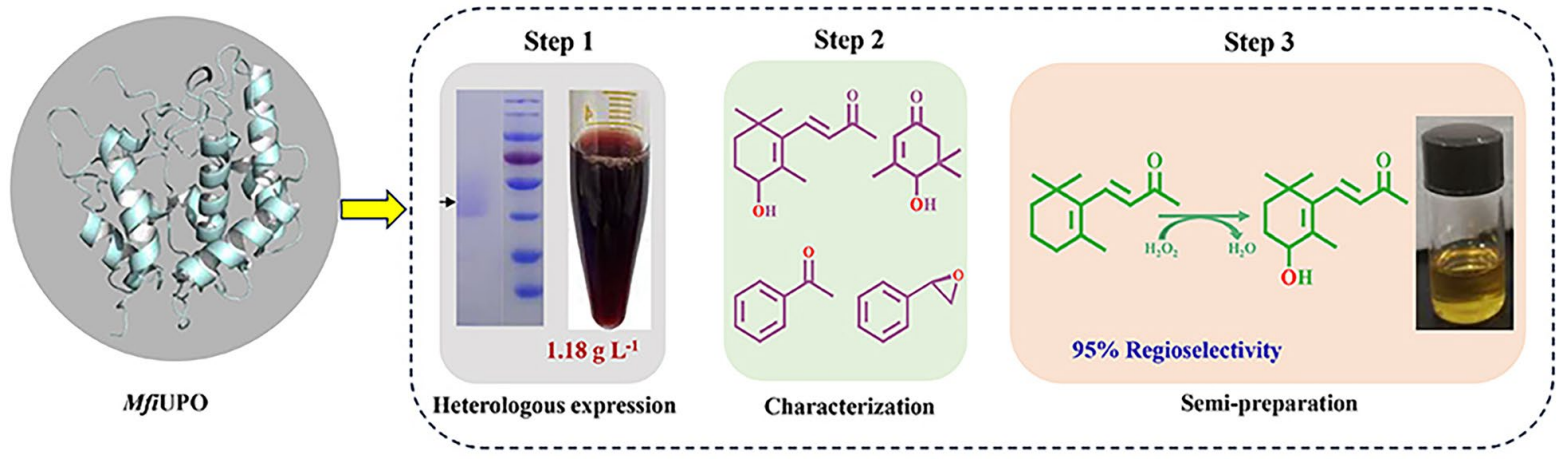

**Supplementary Information:**

The online version contains supplementary material available at 10.1186/s40643-024-00751-x.

## Introduction

Unspecific peroxygenases (UPOs, EC 1.11.2.1) are newly discovered extracellular enzymes which belong to heme-thiolate proteins obtained from fungal species, which catalyze the selective oxyfunctionalization of organic molecules containing inert C-H bond under mild conditions using H_2_O_2_ as oxygen source (Ullrich et al. 2004; Sigmund et al. 2020). Furthermore, the extracellular nature and consequent higher stability, together with their self-sufficient monooxygenase activity (only requiring H_2_O_2_ to be activated), confer enormous advantages to UPOs in biosynthesis (Hobisch et al. 2021).

The initial discovery of the first UPO enzyme occurred in *Agrocybe aegerita* (*Aae*UPO) in 2004 (Ullrich et al. 2004). This significant milestone sparked growing interest in UPOs, which have been designated as the ‘‘generational successors’’ to P450s (Gomez de Santos et al. 2019; Wang et al. 2017). However, despite their advantages of solubility, extracellular nature, stability, and activation by H_2_O_2_, there are several obstacles hindering widespread research, application, and evolution. These include insufficient enzymological information and poor heterologous expression.

Up to now, over 4000 sequences have been annotated as UPOs (Kinner et al. 2021), but there were very few UPOs available with distinct properties such as *Aae*UPO (Molina-Espeja et al. 2014), *Cci*UPO from *Coprinopsis cinerea* (Babot et al. 2013), *Pab*UPO from *Psathyrella aberdarensis* (Gomez de Santos et al. 2021), *Mro*UPO from *Marasmius rotula* (Gröbe et al. 2011), *Cgl*UPO from *Chaetomium globosum* (Kiebist et al. 2017), *Cvi*UPO from *Collariella virescens* (Linde et al. 2020), *Dca*UPO from *Daldinia caldariorum* (Linde et al. 2020), *Hsp*UPO from *Hypoxylon* sp. EC38 (Rotilio et al. 2021), *Mfe*UPO from *Myceliophthora fergusii* and *Mhi*UPO from *Myceliophthora hinnulea* (Püllmann et al. 2021), *Gma*UPO from *Galerina marginata* (Ma et al. 2022), among others. Therefore, it is crucial to access more enzymes to gain further insight into the natural function of UPOs.

Several UPOs have been expressed in their native fungal producers or heterologously in *Aspergillus niger*, *Aspergillus oryzae*, *Escherichia coli*, *P. pastoris* and *Saccharomyces cerevisiae* at the ‘mg-per-liter’ level. The highest protein levels reported so far were obtained from *Mro*UPO with its natural host of *M. rotula* (445 mg L^−1^) and from evolved recombinant r*Aae*UPO produced with *P. pastoris* (217 mg L^−1^) (Gröbe et al. 2011; Molina-Espeja et al. 2015). However, the application in the pharmaceutical, cosmetic and fine chemical industry will only be feasible if cost-effective and reliable heterologous production of recombinant UPOs at the g L^−1^ scale is realized. The low level of heterologous expression for native UPOs is challenging for further characterizations and applications, making it difficult to create new variants with improved properties. Therefore, high level expression is the major challenging task for engineering and application of UPOs on an industrial scale.

In this study, a new unspecific peroxygenase from *M. fiardii* PR910 (*Mfi*UPO) was mined and successfully expressed in *P. pastoris* using its native signal peptide, resulting in production levels at the g L^−1^ scale. The enzymatic properties of r*Mfi*UPO were then investigated, and a variety of diverse substrates were employed for biotransformation. Finally, semipreparative synthesis was conducted on a 100 mL scale by taking the production of 4-OH β-ionone as an example. The obtained results provide a foundation for further study on the practical application and directed evolution of *Mfi*UPO.

## Materials and methods

### Plasmids, strains and chemicals

Plasmid pPICZ-A, strains *P. pastoris* X33 and *E. coli* (DH5α) were purchased from Invitrogen (USA). Restriction endonucleases *Not* I, *Cpo* I, *Spe* I, *BamH* I, *Bgl* II and T4 DNA Ligase were purchased from Takara (Japan). ABTS (2,2ʹ-azinobis (3-ethylbenzothiazoline-6-sulfonic acid) diammonium salt) and NBD (5-nitro-1,3-benzodioxole) used for activity test were purchased from Sigma-Aldrich (USA). All other chemicals and media components were of the highest available purity from Aladdin (China).

### UPO candidate mining

To obtain novel short-type UPOs and evaluate the catalytic activities, *Mro*UPO and *Cgl*UPO were chosen as templates to conduct gene mining from NCBI database. Especially screening was performed by controlling the sequences of amino acids from 200 to 300. Then, 6 candidates annotated as chloroperoxidase and hypothetical protein were selected. The codons of candidates were optimized for *P. pastoris* expression, synthesized by GeneCreate (Wuhan, China), and cloned in the pPICZ-A under the control of the AOX1 promoter through restriction enzymes *Not* I and *Cpo* I. The resulted recombinant plasmid pPICZ-A-UPO was linearized by *Bgl* II and then transformed into *P. pastoris* X33 using electroporation (1.5 kv, 4 ms). The peroxygenase activity of cell-free supernatant was determined using the potential substrate of NBD. The candidate UPO with NBD activity was selected for further research.

### Construction of multiple-copy expression cassette plasmids

The pPICZ-A-*Mfi*UPO contained single copy of target gene was named as pMFI 1. Based on the pMFI 1 plasmid, the additional multiple copies of *Mfi*UPO gene were introduced using the biobrick assembly method (Xiang et al. 2016). pPICZ-A-*Mfi*UPO was digested using *Bgl* II + *Spe* I and *Spe* I + *BamH* I, respectively (Additional file 1: Fig. S1A). The digestive products were ligated by T4 DNA Ligase, then generated the construct pMFI 2 (2 copy). Follow the above method, pMFI 3 (3 copy) and pMFI 4 (4 copy) of the *Mfi*UPO expression cassettes were constructed, respectively. All the multi-copy recombinant plasmids were confirmed by *Bgl* II digestion (Additional file 1: Fig. S1B) and DNA sequencing analysis. The four recombinant plasmids were linearized by *Bgl* II and then transformed into *P. pastoris* X33, respectively, to obtain four different recombinant *P. pastoris* strains MFI1c, MFI2c, MFI3c and MFI4c. Transformants were screened on YPD plates containing 300 mg L^−1^ Zeocin.

### Small-scale flask fermentation of r*Mfi*UPO

Single colonies of recombinant *P. pastoris* were picked and inoculated in 100 mL Buffered minimal glycerol (BMGY) (100 mM potassium phosphate pH 6.0, 1.34% yeast nitrogen base without amino acids, 1% glycerol, 1% yeast extract, 2% tryptone) at 28 ºC and 250 rpm, respectively. When optical density at 600 nm (OD_600_) was above 15, the cell pellets were harvested and resuspended in 100 mL BMMY (100 mM potassium phosphate buffer pH 6.0, 1.34% yeast nitrogen base without amino acids, 4 × 10^–5^% biotin, 1% methanol, 1% yeast extract, 2% tryptone) in a 1000 mL conical flask at 25 ºC and 250 rpm. They were supplemented with 1% (v/v) methanol every 24 h. After 168 h induction, the culture was harvested by centrifugation and supernatant was used for NBD activity determination. The clone with the highest activity was selected for cultivation in bioreactor.

### Production of r*Mfi*UPO in 5-L fed-batch bioreactor

*P. pastoris* clone containing *Mfi*UPO was cultivated in 5 L glass vessel bioreactor (T&J Bio-engineering, Shanghai, China) and performed according to the *Pichia* Fermentation Process Guidelines of Invitrogen. 100 mL of preculture grown on YPD medium at 220 rpm and 28 ºC for 24 h was added into the 2.5 L basal salts medium (BSM) and run at 500 rpm and 28 ºC. After the glycerol has been completely consumed, the glycerol fed-batch phase followed with the addition of 50% (w/v) glycerol feed containing 12 mL L^−1^ of PTM1 trace salt (dissolved oxygen (DO) concentration should be kept above 30%) until the OD_600_ was above 180. The methanol feed was started by adding 5 mL h^−1^ per liter fermentation volume, without containing PTM1 trace salt. Within the first two to three hours, the addition of methanol was slowly increased so that the cells could adapt and the DO spike stay above 30%. Samples were taken regularly and NBD activity was determined. When the enzyme activity did not continue to increase, the cell-free supernatant containing r*Mfi*UPO was harvested by centrifugation at 16,000 rpm for 10 min at 4 ºC.

### Purification of r*Mfi*UPO

The cell-free supernatant was concentrated 20-fold by tangential flow ultrafiltration (cut-off 10 kDa), then applied to anion exchange chromatography using 10 mM sodium acetate pH 5.5 as mobile phase eluting with a 0.67 M NaCl gradient within 15 column volumes. Fractions with NBD activities were pooled, further concentrated and desalinated by ultrafiltration. Enzyme concentration was estimated according to the characteristic UV–vis band of the reduced UPO complex (Fe^2+^-heme) with carbon monoxide (Otey et al. 2003).

### Activity assays

r*Mfi*UPO activities were determined by absorbance-based methods using ABTS, NBD and veratryl alcohol (VA) as substrates. Activities assays were determined according to the methods (Kiebist et al. 2017; Poraj-Kobielska et al. 2012; Ullrich et al. 2004).

- (i) ABTS. 100 μL cell-free supernatant was mixed with 890 μL of 100 mM sodium phosphate/citrate buffer (pH 4.4) containing 0.3 mM ABTS and 2 mM H_2_O_2_. The plates were briefly stirred, and the absorbance was measured at 418 nm (ε_418_ = 36,000 M^−1^ cm^−1^).
- (ii) NBD. 100 μL cell-free supernatant was mixed with 890 μL of 100 mM sodium phosphate buffer (pH 7.0) containing 1 mM NBD (final concentration of acetonitrile, 15%) and 1 mM H_2_O_2_. The plates were briefly stirred, and the absorbance was measured at 425 nm (ε_425_ = 9700 M^−1^ cm^−1^).
- (iii) VA. 100 μL cell-free supernatant was mixed with 890 μL of 100 mM sodium phosphate buffer (pH 7.0) containing 5 mM VA and 1 mM H_2_O_2_. The plates were briefly stirred, and the absorbance was measured at 310 nm (ε_310_ = 9300 M^−1^ cm^−1^).

### Enzyme characterization

#### pH activity profiles

The pH optima of r*Mfi*UPO were determined for ABTS, NBD and VA, respectively. Reactions at appropriate concentrations of ABTS (300 μM), NBD (500 μM) and VA (5 mM) were analyzed in citric acid/dibasic sodium phosphate buffer (pH 2.2 ~ 5.0) and potassium phosphate (pH 5.5 ~ 10.0) buffers. Formation of the ABTS cation radical (ɛ_418_, 36,000 M^−1^ cm^−1^), 4-nitrocatechol (ɛ_425_, 9700 M^−1^ cm^−1^) and veratraldehyde (ɛ_310_, 9300 M^−1^ cm^−1^) products were tested by spectrophotometer, respectively.

#### Thermostability and solvent sensitivity

r*Mfi*UPO (final concentration 0.5 μM) was prepared in 50 mM potassium phosphate buffer (pH 7.0), then splited into aliquotes of 50 μL. Sample was incubated in a thermocycler on a gradient ranging from 40 °C to 90 °C for 10 min, then chilled on ice and incubated at room temperature. Finally, samples were subjected to the ABTS and NBD assays as described above for activity assay. Thermostability values were calculated from the ratios between the residual activities at different temperatures and the initial activity at room temperature.

The relative activities in organic solvents were assessed with the ABTS activity assay, supplementing with 5% ~ 30% concentration of organic solvent (acetone, acetonitrile, DMSO, methanol and ethanol). Tolerance in organic solvent (retained activity after incubating 50 nM enzyme with 50% solvents after 5, 15 and 40 h, respectively) was defined as the ratio of the activity in the presence of organic solvent to that in the absence of organic solvent (control).

#### Enzyme kinetics

Kinetic constants were estimated at the optimal pH. Kinetic curves were obtained by varying the concentrations of ABTS (from 0.07 μM to 0.3 mM), NBD (from 0.07 μM to 1.5 mM) and VA (from 0.07 μM to 4.5 mM) in spectrophotometer and adding 3 ~ 75 nM r*Mfi*UPO, respectively. The reactions were initiated by adding 2 mM H_2_O_2_, the time was monitored to ensure that the rates of reactions were in linear phase, and values were calculated as the change in absorbance over time. All reactions were incubated at 30 °C in triplicate. Data were fitted to the Michaelis–Menten equation using GraphPad Prism software 7.0.

#### SDS-PAGE analysis and deglycosylation

The molecular mass of r*Mfi*UPO was analyzed by sodium dodecyl sulfate–polyacrylamide gel electrophoresis (SDS-PAGE) on a 12% gel. The separated protein bands were visualized with Coomassie Brilliant Blue R-250 staining, a protein molecular weight marker was used as standard. Enzymatic deglycosylation was performed using Endo-β-N-acetylglucosaminidase H (Endo H) for 12 h at 37 °C and detected by SDS-PAGE.

### Bioconversions

The hydroxylation reaction was carried out in triplicate in glass vials at 30 °C and 300 rpm with a final volume of 1 mL. The reaction mixtures contained purified r*Mfi*UPO (0.25 μM) solution, phosphate buffer (50 mM) pH 6.0, and substrates (**1a** ~ **3a** and **5a**, 10 mM). The reaction was started by the continuous addition of H_2_O_2_ to the final concentration of 2 mM. After 12 h conversions, samples containing substrates **1a** ~ **3a** and **5a** were extracted with ethyl acetate (2 × 2 mL), and the organic layer was dried over Na_2_SO_4_, and analyzed on gas chromatography (GC). The detailed methods for GC analysis were described in the Additional file 1: Table S1.

The epoxidation reactions (1 mL scale) were performed as biological triplicates in 100 mM potassium phosphate (pH 7.0) containing 0.25 μM of r*Mfi*UPO, 1 mM of styrene **4a** and 2 mM H_2_O_2_. The substrate was prior dissolved in pure acetone yielding a 10% (v/v) co-solvent ratio in the final reaction mixture. Reactions were performed for 12 h at 30 °C in vials constantly shaken at 300 rpm, and subsequently quenched by the addition of an equal volume of ethyl acetate. Extraction was accomplished by vigorous vortexing for 30 s, followed by brief centrifugation (12,000 rpm for 1 min). The separated organic layer was then used for GC analysis (Additional file 1: Table S1).

### Semipreparative-scale biotransformation

Synthesis of **3b**: The reaction was carried out on a 100 mL scale in a wave vial with a septum cap at 30 °C and 300 rpm. The reaction mixture contained r*Mfi*UPO (c final = 16 μM) and the substrate β-ionone **3a** (2.88 g, c final = 150 mM) in phosphate buffer (50 mM, pH 6.0) with 15 vol % acetone. The conversion was stirred at room temperature while H_2_O_2_ (4 mM h^−1^) was continuously supplied with a syringe pump. Samples (2 mL) were taken from the reaction mixture every 30 min and ethyl acetate (2 mL) was added. The samples were centrifuged, and the supernatants were analyzed by GC. After complete conversion, the reaction mixture was extracted (3 × 200 mL ethyl acetate), then the combined organic phases were dried over Na_2_SO_4_, and evaporated under reduced pressure, and the product was purified by chromatography on silica gel with ethyl acetate/petroleum ether (1:10) as the eluent. The structure of preparative-scale biotransformation product **3b** was verified by NMR.

## Results and discussion

### Unspecific peroxygenase candidate mining

**Fig. 1:**
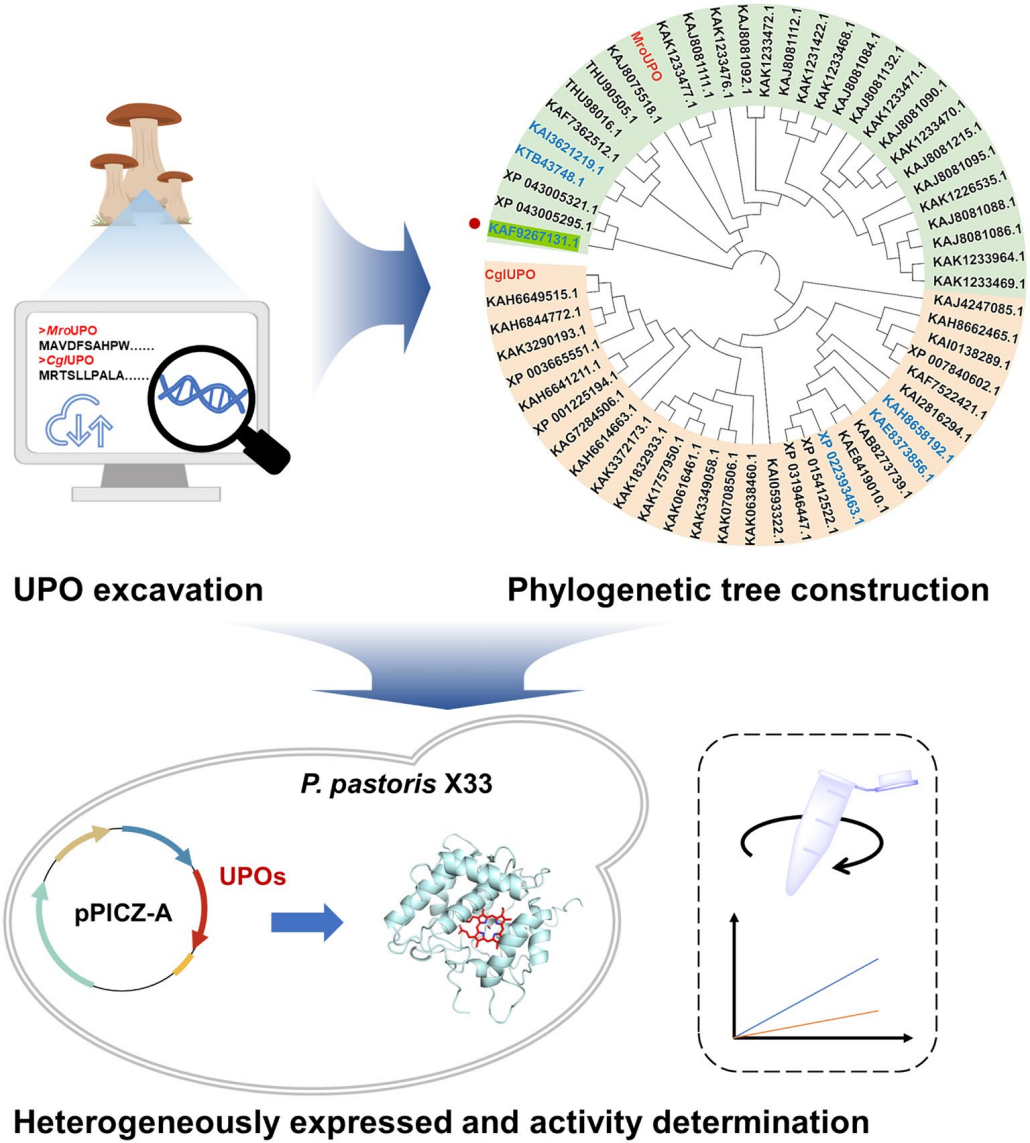
UPO excavation, phylogenetic tree construction and heterogeneous expression and activity determination. Phylogenetic tree of 60 putative UPO candidate sequences was generated using probes *Mro*UPO and *Cgl*UPO (shown in red), of which 6 candidates were selected for heterologous expression in *P. pastoris* (shown in blue)

To identify novel efficient short-UPOs from the large amounts of putative sequences of NCBI database, *Mro*UPO and *Cgl*UPO were chosen as prototype enzymes to facilitate target sequence selection. Firstly, according to multiple sequence alignments, 60 sequences were selected for generating a phylogenetic tree based on its high sequence identity (> 50%) (Fig. 1). Secondly, based on existing knowledge from literature combined with homology models of UPOs, six enzyme candidates were selected (Fig. 1, shown in blue). The sequence-optimized genes were synthesized and cloned into pPICZ-A plasmid, then linearized recombinant plasmids were transformed into *P. pastoris* X33 and target proteins were induced with methanol. The peroxygenase activities of six UPOs were determined using NBD as the model substrate. The results showed that only the *Mfi*UPO (accession code, KAF9267131.1, amino acid sequence see Additional file 1: Table S2) from *M. fiardii* PR910 exhibited activity (457 U L^−1^) when expressed on shaking flask, while other enzymes were completely inactive.

### Optimization of heterologous expression of r*Mfi*UPO

**Fig. 2:**
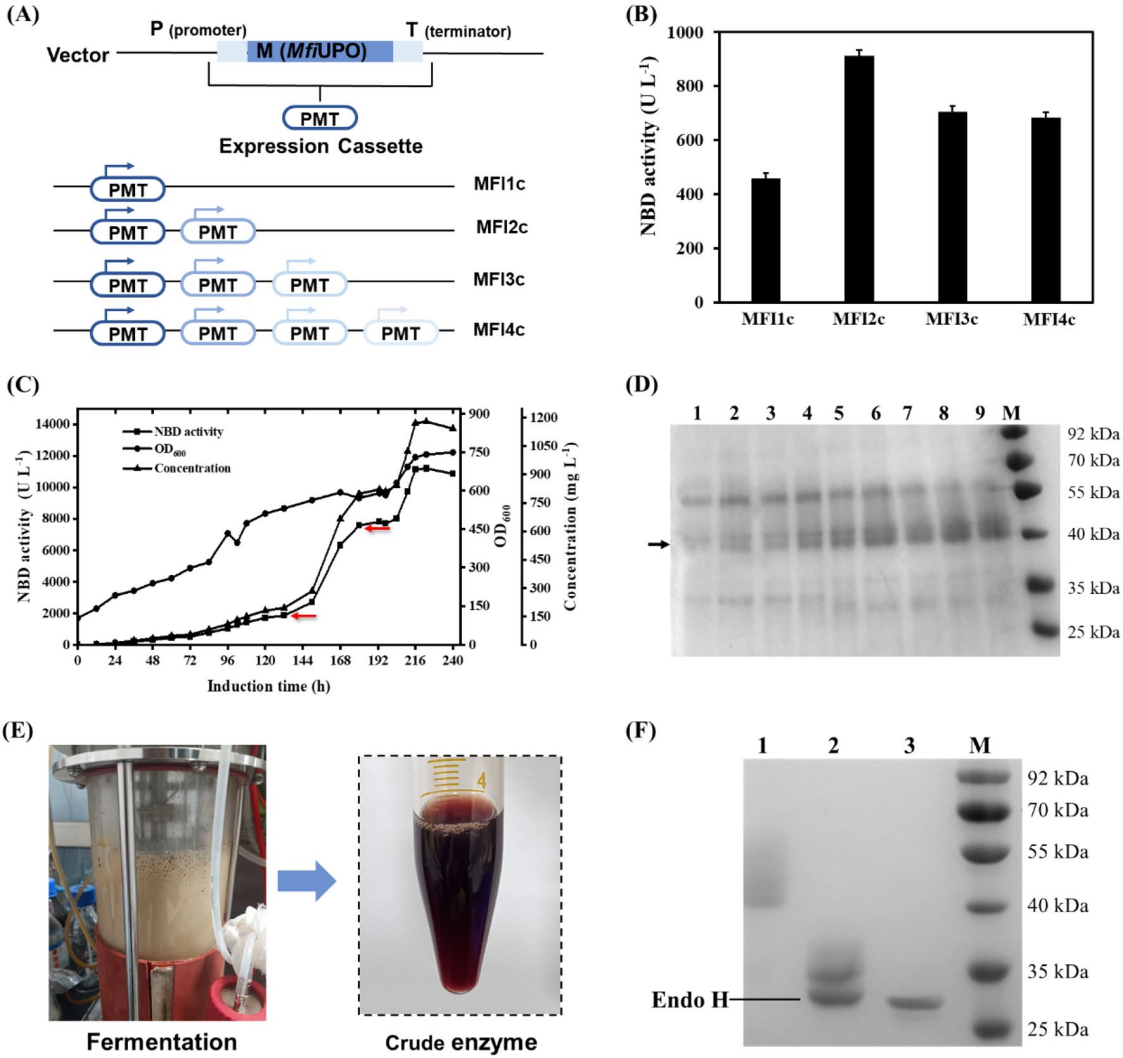
**A** Strategy of multi-copy strains construction. **B** NBD activities of multi-copy strains on shaking flask scale. **C** The high-cell-density fermentation of *P. pastoris* MFI2c in a 5 L bioreactor. The red arrow showed that 0.5% (m/v) yeast extract and 1.0% (m/v) tryptone were collaboratively supplemented at that time. **D** SDS-PAGE analysis of interval sampling.1–9 were the samples of 30 h, 60 h, 84 h, 108 h, 146 h, 180 h, 200 h, 210 h and 220 h. **E** Supernatant after centrifugation. **F** Assays of purified and N-deglycosylation r*Mfi*UPO. 1, Purified r*Mfi*UPO; 2, N-deglycosylation r*Mfi*UPO and Endo H; 3, Endo H

To improve the expression of r*Mfi*UPO, biobrick assembly method was adopted to construct multiple-copy plasmids (Fig. 2A), the resulted plasmids were then linearized and transformed into *P. pastoris* X33, respectively. The positive clones of four recombinant *P. pastoris* strains MFI1c, MFI2c, MFI3c and MFI4c (means single-copy, 2-copy, 3-copy and 4-copy, respectively) were selected and expressed on shaking flask scale, respectively. After 7 d methanol induction, the results showed that the activities of multiple-copy expression cassette were better than that of single-copy, while the expression level of three- and four-copy were lower than that of two-copy, which needs further investigation. The NBD activities of r*Mfi*UPO in recombinant strains MFI1c, MFI2c, MFI3c and MFI4c were 457 U L^−1^, 912 U L^−1^, 705 U L^−1^ and 685 U L^−1^, respectively (Fig. 2B). Additionally, the pHBM905M vector was also used for the construction of *Mfi*UPO multiple-copy expression according to the procedure reported previously (Song et al. 2020). However, the activities of recombinant strains were lower than that of pPICZ-A vector (Additional file 1: Table S3).

The recombinant strain MFI2c was then selected for r*Mfi*UPO production in a 5 L bioreactor, after 223 h methanol induction, the final OD_600_, NBD activity and protein concentration were 741, 11189 U L^−1^ and 1180 mg L^−1^, respectively (Fig. 2C). The expression level of r*Mfi*UPO reached 1.18 g L^−1^, which was the highest heterologous expression level of UPOs in *P. pastoris* up to now (Table 1). It needs to be pointed out that yeast extract and tryptone were significant factors for r*Mfi*UPO expression, during the production, 0.5% (m/v) yeast extract and 1.0% (m/v) tryptone were collaboratively supplemented in 130 h and 180 h, respectively (Fig. 2C, red arrow). The SDS-PAGE analysis of interval sampling indicated that the target protein was gradually increased (Fig. 2D). Additionally, the reddish supernatant from 5 L bioreactor also showed high protein concentration of r*Mfi*UPO (Fig. 2E). Purified r*Mfi*UPO revealed a smeared band in the range of 40 to 55 kDa, and exhibited a band at approx. 35 kDa that was retained after N-deglycosylation with Endo H (Fig. 2F). To understand how glycosylation affects enzymatic activity, we tested the activity of deglycosylated r*Mfi*UPO and compared it to r*Mfi*UPO (Additional file 1: Fig. S2). The results showed that the deglycosylated enzyme had a slightly lower activity, with a 16.6% reduction compared to r*Mfi*UPO.

**Table 1:** Comparison of different UPO expression using *P. pastoris*

UPO	Culture volume (L)	Total protein (mg L^−1^)	References
*rMfi*UPO	5.0	1180	This study
*Pab*UPO-II	5.0	290	(Gomez de Santos et al. 2021)
*Hsp*UPO	5.0	200	(Rotilio et al. 2021)
*Aae*UPO PaDa-I	7.0	217	(Molina-Espeja et al. 2015)
*Cgl*UPO	Shake flask	9	(Püllmann et al. 2021a)
*Mfe*UPO, *Mhi*UPO, *Tte*UPO, *Mth*UPO	Shake flask	6.5 5.7 21.9 22.4	(Püllmann et al. 2021b)
*Abr*UPO	7.5	742	(Schmitz et al. 2023)

### Characterization of r*Mfi*UPO

**Fig. 3:**
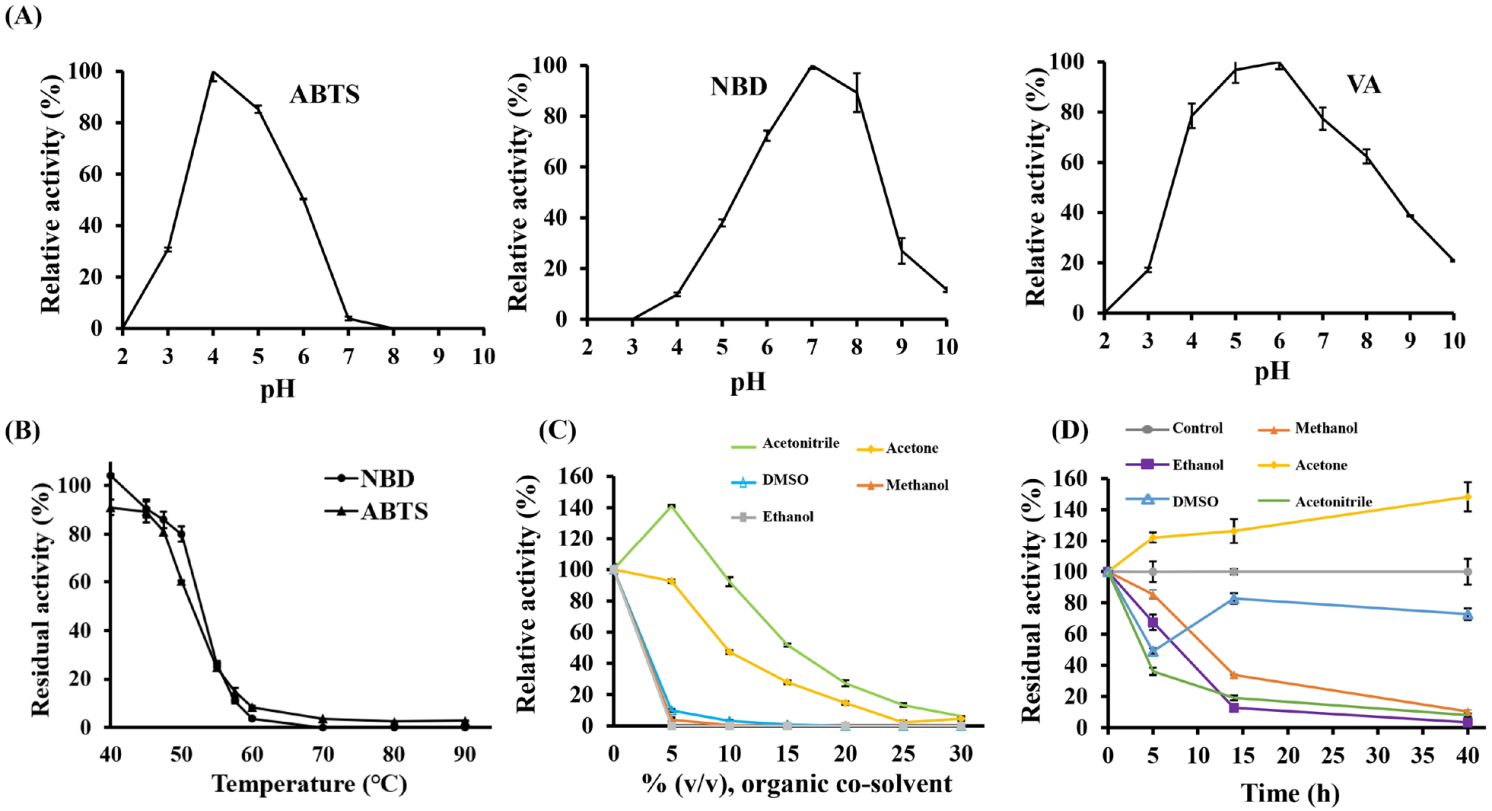
**A** pH activity profiles of r*Mfi*UPO. Reactions were analyzed in citric acid/dibasic sodium phosphate buffer (pH 2.2 ~ 5.0) and potassium phosphate (pH 5.5 ~ 10.0) buffers. **B** Thermostability of r*Mfi*UPO towards ABTS and NBD. Each point represents the mean and standard deviation of 3 independent experiments. **C** The relative activities of r*Mfi*UPO in organic cosolvents were assessed with 2 mM H_2_O_2_ and 0.3 mM ABTS in 100 mM sodium phosphate/citrate buffer (pH 4.0) containing the corresponding concentration of cosolvent. **D** The stabilities of r*Mfi*UPO after incubation for 40 h in 50% organic cosolvents were assessed by incubating enzyme samples in 100 mM potassium phosphate buffer (pH 7.0) containing 50% (vol/vol) organic cosolvent in screw-cap vials. After 40 h, aliquots were removed and analyzed in an activity assay with 2 mM H_2_O_2_ and 0.3 mM ABTS in 100 mM sodium phosphate/citrate buffer (pH 4.0)

The pH value was a critical factor on bioconversions, r*Mfi*UPO was optimally active at pH 7.0 and 6.0 on substrates NBD and VA, respectively, whereas acidic pH 4.0 was optimal for oxidation of substrate ABTS (Fig. 3A). Below 50 °C, r*Mfi*UPO was only slightly affected, but when the temperature exceeded 60 °C, it was mostly inactivated. (Fig. 3B).

For the investigation of effect of organic solvents, several organic solvents such as acetone, acetonitrile, DMSO, methanol and ethanol were used to improve the availability of hydrophobic substrates in the aqueous phase. The activity and stability of r*Mfi*UPO were evaluated in the presence of high concentrations of cosolvents with different polarities. Activity was reduced drastically in the presence of increasing concentrations of cosolvents in the following order: ethanol > methanol > DMSO > acetone > acetonitrile (Fig. 3C). In terms of stability in cosolvents, r*Mfi*UPO was stable at concentrations as high as 50% (vol/vol) towards acetone and DMSO, with a half-life of over 40 h. Furthermore, the activity was boosted by acetone, which was up to 150% over 40 h (Fig. 3D). The reason for this could be attributed to the enhanced solubility of the substrate at higher acetone concentrations, which in turn increases the enzyme’s affinity for the substrate. It is worth noting that the enhanced enzyme activity resulting from the addition of acetone is a common occurrence, as reported in several other UPOs (Peter et al. 2011; Babot et al. 2013; Gomez de Santos et al. 2023).

**Fig. 4:**
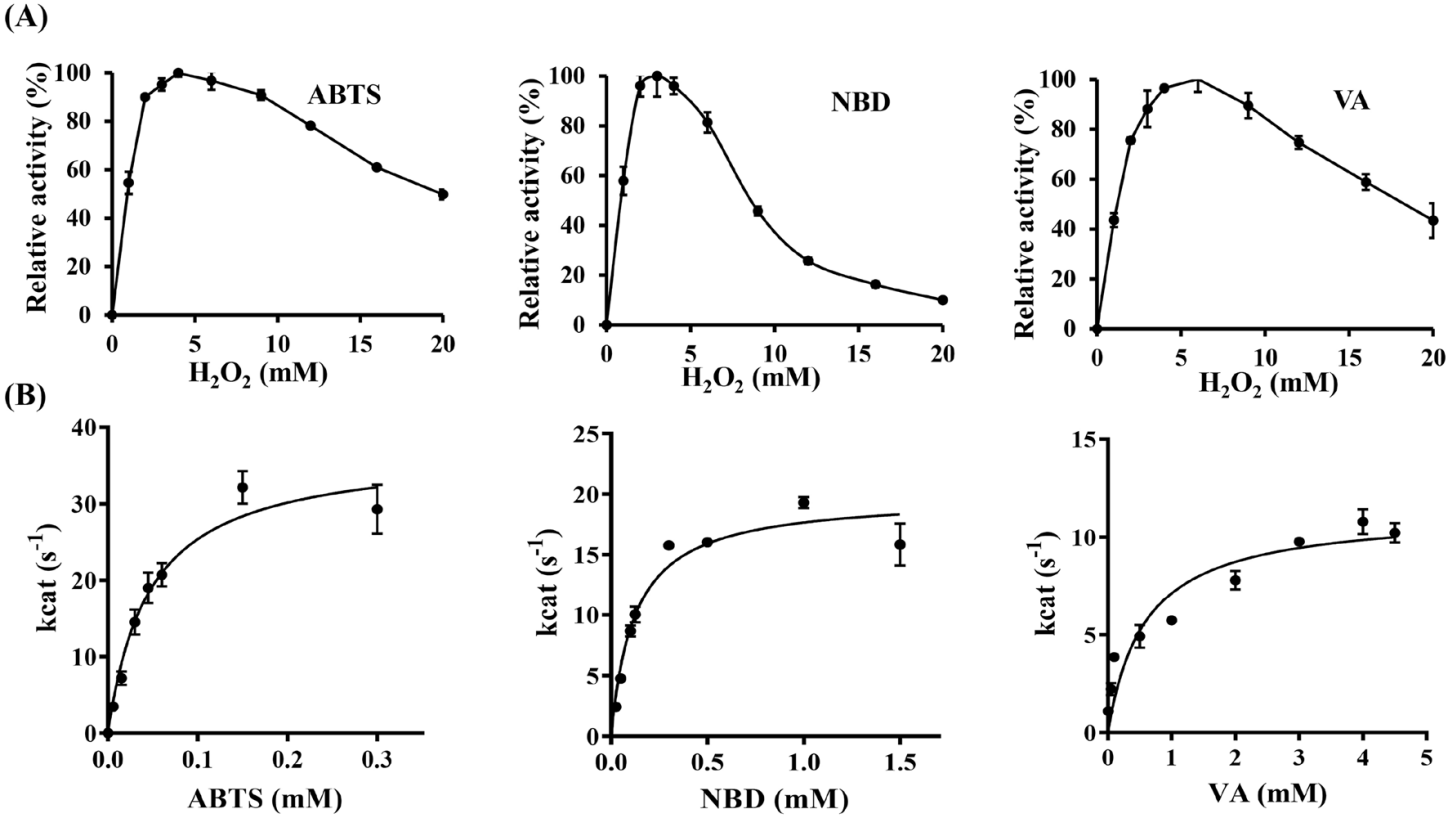
**A** H_2_O_2_ sensitivity of r*Mfi*UPO. Assay mixtures were consisted of 50 mM sodium citrate (pH 4.5), 30 μM ABTS, 50 nM r*Mfi*UPO, and different concentration of H_2_O_2_; 50 mM potassium phosphate (pH 7.0), 0.5 mM NBD, 50 nM r*Mfi*UPO and different concentration of H_2_O_2_ and 50 mM potassium phosphate (pH 6.0), 2 mM VA, 100 nM r*Mfi*UPO and different concentration of H_2_O_2_, respectively. **B** Michaelis–Menten curves were observed for ABTS, NBD and VA. The assay mixtures were consisted of 50 mM sodium citrate (pH 4.5), 30 μM ABTS, 50 nM r*Mfi*UPO and 2 mM H_2_O_2_; 50 mM potassium phosphate (pH 7.0), 0.5 mM NBD, 50 nM r*Mfi*UPO and 2 mM H_2_O_2_; 50 mM potassium phosphate (pH 6.0), 2 mM VA, 100 nM r*Mfi*UPO and 2 mM H_2_O_2_, respectively

To avoid H_2_O_2_ oxidative damage on enzyme, the capacity of r*Mfi*UPO withstand various concentrations of H_2_O_2_ upon prolonged incubation was determined. The experiments were conducted by employing r*Mfi*UPO with substrates ABTS, NBD and VA. As shown in Fig. 4A, the optimal activities of r*Mfi*UPO for ABTS and NBD were observed at 4.0 and 3.8 mM H_2_O_2_, respectively, whereas for substrate VA, the highest activity was observed at 6.0 mM H_2_O_2_. The results shown that biotransformations catalyzed by r*Mfi*UPO were less affected by elevated concentrations of H_2_O_2_, which also had certain reference significance for maintaining H_2_O_2_ concentrations below 4 mM during oxidation reaction.

The Michealis Mentens constants (*K*_m_), catalytic constants (*k*_cat_) and catalytic efficiencies ratios (*k*_cat_/*K*_m_) of ABTS, NBD and VA substrates were summarized in Fig. 4B and Table 2. r*Mfi*UPO efficiently oxidized towards the non-phenolic substrate ABTS, exhibited remarkably low *K*_m_ value of 22 μM, and the activity reached the plateau (*k*_cat_ = 33.05 ± 2.48) at about 250 μM concentration. As a result of a lower measured *K*_m_, r*Mfi*UPO had a better binding affinity with ABTS as compared to *Aae*UPO (25 μM), *Hsp*UPO (30 μM), PaDa-I (48 μM), r*Dca*UPO (59 μM), *Mro*UPO (71 μM), *Pab*UPO-I (105 μM), *Pab*UPO-II (128 μM), *Cgl*UPO (106 μM) and r*Cvi*UPO (239 μM). Furthermore, the binding affinity with substrate NBD (*K*_m_ 93 μM) was also higher than *Aae*UPO (684 μM), PaDa-I (483 μM) and *Cgl*UPO (532 μM) (Molina-Espeja et al. 2014; Gröbe et al. 2011; Rotilio et al. 2021; Gomez de Santos et al. 2021; Linde et al. 2020; Kiebist et al. 2017).

**Table 2:** Steady-State kinetic parameters of r*Mfi*UPO

Substrate	*K*_m_ (mM)	*k*_cat_ (s^−1^)	*k*_cat_/*K*_m_ (s^−1^ mM^−1^)
ABTS	0.022 ± 0.007	33.05 ± 2.48	1495.5
NBD	0.093 ± 0.014	17.68 ± 0.72	190.0
VA	0.392 ± 0.109	11.31 ± 0.61	28.9

For each substrate, reactions were performed in triplicate, with monitoring of the increases in absorption for ABTS (ε_418_ = 36,000 M^−1^ cm^−1^), NBD (ε_425_ = 9700 M^−1^ cm^−1^) and veratryl alcohol (ε_310_ = 9300 M^−1^ cm^−1^)

### Reaction potential of r*Mfi*UPO

To characterize the oxidation capacity of r*Mfi*UPO, various substrates were investigated (Table 3). Ethylbenzene **1a**, a common substrate for hydroxylation test, was regioselectively oxidized at the benzylic position to generate the corresponding (*R*)-alcohol **1b** with 63% *ee* (Entry 1). In addition, phenol and β-ionone were also chosen as substrates for the hydroxylation reaction. Phenol **2a** was selectively oxidized to hydroquinone **2b** and catechol **2c** in 80% and 20% yields, respectively (Entry 2). β-ionone **3a**, a common terpenoid which has been explored by UPOs in recent years, could be oxidized efficiently by r*Mfi*UPO to give 4-OH-β-ionone **3b** with high regioselectivity (95%) (Entry 3). Turning to alkene, the epoxidation of styrene **4a** resulted in complete conversion, yielding corresponding styrene oxides with only 7.1% *ee* (*R*) (Entry 4). Isophorone **5a** was oxidized to produce 4-hydroxyisophoron **5b,** 2,2,6-trimethylcyclo hexane-1,4-dione ketone **5c** and 2,3-isophorone epoxide **5d** with yields of 53%, 7% and 33%, respectively (Entry 5). The formation of the corresponding ketone **1d** and **5c** indicated that r*Mfi*UPO has also the potential to perform the alcohol oxidation. Detailed information on the structural assignment of the products were showed in Additional file 1: Figs. S3, S4, S5, S6.

**Table 3:**
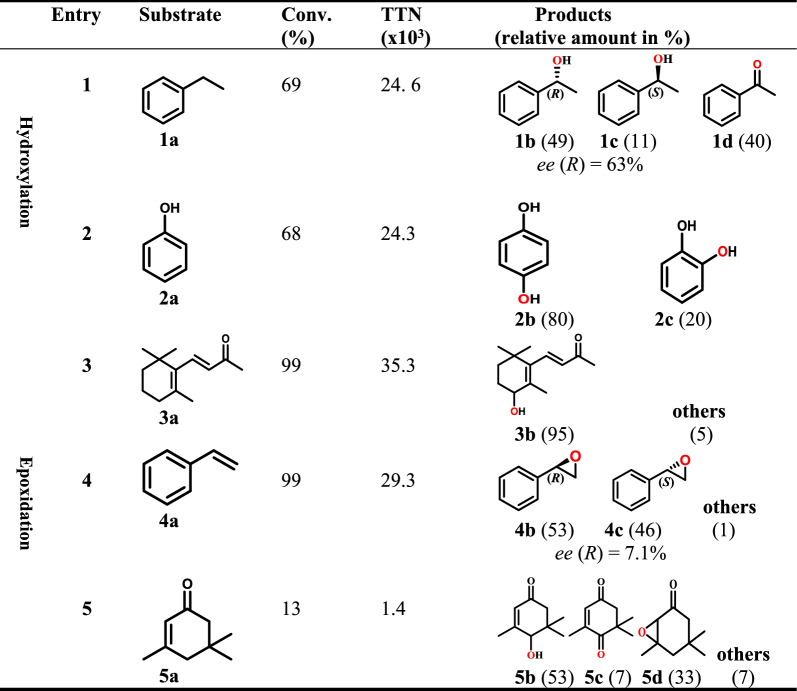
Results of biotransformations catalyzed by r*Mfi*UPO

1a, ethylbenzene; 1b, (R)-1-phenylethanol; 1c, (S)-1-phenylethanol; 1d, acetophenone; 2a, phenol; 2b, hydroquinone; 2c, catechol; 3a, β-ionone; 3b, 4-OH-β-ionone; 4a, styrene; 4b, (R)-styrene oxide; 4c, (S)-styrene oxide; 5a, isophorone; 5b, 4-hydroxyisophoron; 5c, 2,2,6-trimethylcyclo hexane-1,4-dione ketone; 5d, 2,3-isophorone epoxide

It needs to be pointed out that the regioselectvity of r*Mfi*UPO catalyzed hydroxylation of β-ionone at C-4 position represents the highest reported among current UPOs. Both *Cgl*UPO and *Hin*UPO were able to oxygenate β-ionone to give 4-OH β-ionone as main product, but with lower regioselectivity (84% and 79%) relative to r*Mfi*UPO. Moreover, for *Aae*UPO, *Mro*UPO, *Cci*UPO and *Dca*UPO, in addition to C-4 hydroxylation, other positions (C-2 and C-3) hydroxylated products were also observed, which led to the much lower regioselectivity with the values ranging from 56 to 63% (Table 4). Thus, the highest regioselectivity of r*Mfi*UPO makes it particularly advantageous for the efficient preparation and isolation of 4-OH β-ionone. It was known that the 4-OH β-ionones are the main aroma components of floral scents and important intermediates in the synthesis of hormone abscisic acid, which are particularly attractive for the flavor and fragrance industry and chemical synthesis (Brenna et al. 2002; Larroche et al. 1995).

**Table 4:** Comparation of oxidation position of β-ionone by different UPOs

UPO	Products (%)						References
	4-OH-β-Ionone	3-OH-β-Ionone	2-OH-β- Ionone	10-OH-β-Ionone	13-OH-β-Ionone	Others	
r*Mfi*UPO	95					5	This study
*Cgl*UPO	84		1			15	(Babot et al. 2020)
*Hin*UPO	79					21	
*Aae*UPO	58	22	13			7	
*Mro*UPO	56		25			19	
*Cci*UPO	63	19	2	14		2	
*Dca*UPO	58	19	3		7	13	

**Fig. 5:**
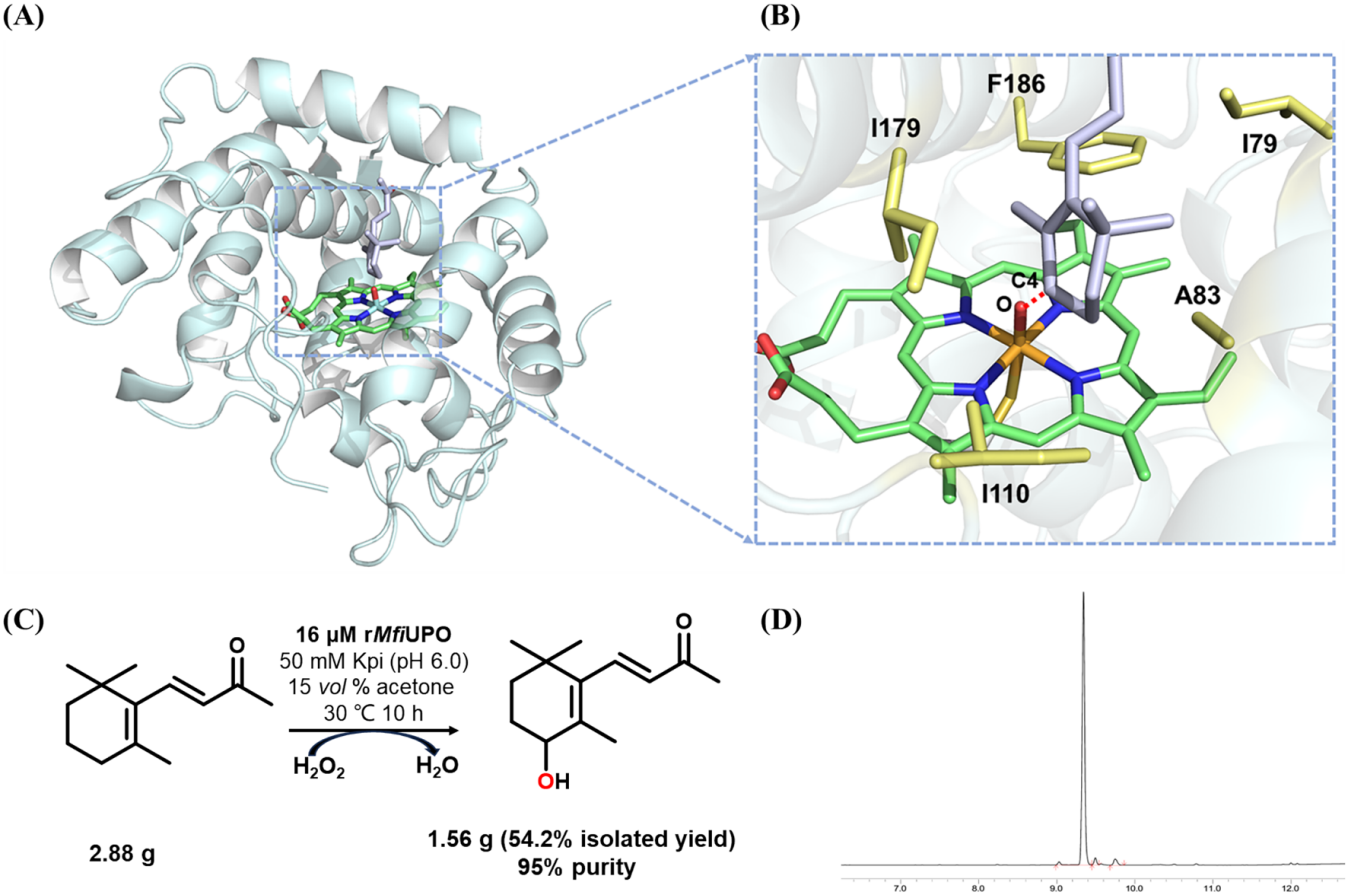
Analysis of the semi-preparative production of 4-OH-β-ionone. **A** Predicted structure of *rMfi*UPO (cyan) with heme (green). **B** Molecular Docking analysis of r*Mfi*UPO with β-ionone. The substrate, heme and substrate binding residues were colored in light purple, green and yellow, respectively. **C** Semi-preparative scale reactions of β-ionone (150 mM) by r*Mfi*UPO (16 μM) with continuously supplied H_2_O_2_ (c total = 110 mM) in a 100 mL scale. **D** GC analysis of 4-OH-β-ionone

Next, to reveal the molecular basis of the high regioselectivity of r*Mfi*UPO for C4 hydroxylation of β-ionone, we performed structural and computational analyses. First, Alphafold2 was used to predict the 3D structure of r*Mfi*UPO. As shown in Fig. 5A, r*Mfi*UPO is mainly composed of 8 α-helices and several loops. The substrate β-ionone was then docked to the active site to simulate its hydroxylation reaction (Fig. 5B). It can be seen that C4 is the closest to the Fe = O bond of r*Mfi*UPO, making it easy to complete monooxygenation at the C4 position. This is consistent with the experiment results (Table 4). In addition, its binding pocket is composed of multiple hydrophobic residues and is highly hydrophobic. In particular, I179 and I110 have a strong anchoring effect on the hydrophobic ring of β-ionone, thus shortening the distance between C4 and heme. Finally, to demonstrate the practical feasibility of r*Mfi*UPO catalyzed reaction, a semi-preparative production of 4-OH-β-ionone on a 100 mL scale was carried out with β-ionone as a substrate. As a result, the upscaled reaction (30 °C, 10 h) led to the synthesis of 1.56 g 4-OH-β-ionone (**3b**) (54.2% isolated yield) with 95% purity (Fig. 5C, D), indicating a great potential for industrial application. The structure of 4-OH-β-ionone (**3b**) was verified by NMR (Additional file 1: Fig. S7).

## Conclusions

To sum up, a novel r*Mfi*UPO was mined using *Mro*UPO as a probe. Its heterologous expression in *P. pastoris* reached 1.18 g L^−1^, marking the highest record for UPO production to date. r*Mfi*UPO exhibited lower *K*_m_ values for both peroxidase and peroxygenase reactions and demonstrated exceptional oxidation characteristics. Notably, it oxidized β-ionone to produce 4-OH-β-ionone with a remarkable regioselectivity of 95%, surpassing the performance of currently reported UPOs. With its high-level expression, r*Mfi*UPO could serve as a new template for the rational design of variants to achieve desirable traits such as increased activity, stability, a broader substrate spectrum, and higher regio- and enantioselectivity. This could pave the way for an efficient oxyfunctionalization biocatalyst in synthesizing high-value chemicals for industrial production applications.

### Supplementary Information

**Additional file 1: Table S1.** GC analytical methods. **Table S2.** Amino acid sequence of *Mfi*UPO (accession code, KAF9267131.1) from *M. fiardii *PR910. **Table S3.** The NBD activities of r*Mfi*UPO in recombinant strains constructed with pPICZ-A and pHBM905M vectors, respectively. **Fig. S1.** Construction and confirmation of multi-copy expression cassette plasmid. **Fig. S2.** Activity determination of r*Mfi*UPO and deglycosylated r*Mfi*UPO. **Fig. S3.** GC analysis of ethylbenzene and conversion products. **Fig. S4.** GC analysis of phenol and conversion products. **Fig S5.** GC analysis of styrene and conversion products. **Fig. S6.** GC/GC-MS analysis of isophorone and conversion products. **Fig. S7.** NMR spectra of 4-OH-β-ionone (**3b**), isolated from semi-preparative scale biotransformation.

## Data Availability

All data generated or analyzed during this study are included in this published article.
