# Supplementary material for: Identification, heterologous expression and characterization of a new unspecific peroxygenase from Marasmius fiardii PR-910
Source: Bioresour Bioprocess. 2024 Mar 27;11(1):33. doi: 10.1186/s40643-024-00751-x (PMC10992195; doi:10.1186/s40643-024-00751-x)
Supplement: Supplementary file 1 — Additional file 1: Table S1. GC analytical methods. Table S2. Amino acid sequence of MfiUPO (accession code, KAF9267131.1) from M. fiardii PR910. Table S3. The NBD activities of rMfiUPO in recombinant strains constructed with pPICZ-A and pHBM905M vectors, respectively. Fig. S1. Construction and confirmation of multi-copy expression cassette plasmid. Fig. S2. Activity determination of rMfiUPO and deglycosylated rMfiUPO. Fig. S3. GC analysis of ethylbenzene and conversion products. Fig. S4. GC analysis of phenol and conversion products. Fig S5. GC analysis of styrene and conversion products. Fig. S6. GC/GC-MS analysis of isophorone and conversion products. Fig. S7. NMR spectra of 4-OH-β-ionone (3b), isolated from semi-preparative scale biotransformation. [file 40643_2024_751_MOESM1_ESM.docx]

Supporting information

**Identification, Heterologous Expression and Characterization of a New Unspecific Peroxygenase from *Marasmius fiardii* PR-910**

Xin Fu, Kexin Lin, Xiaodong Zhang, Zhiyong Guo, Lixin Kang*, Aitao Li*

State Key Laboratory of Biocatalysis and Enzyme Engineering, Hubei Key Laboratory of Industrial Biotechnology, School of Life Sciences, Hubei University, #368 Youyi Road, Wuhan, 430062 P.R. China.

*Correspondence: Lixin Kang, lixinkang2007@hubu.edu.cn; Aitao Li, aitaoli@hubu.edu.cn

**Table of Contents**

**1 Table S1**................................................................................................................................2

**2 Table S2**.................................................................................................................................2

**3 Table S3**.................................................................................................................................2

**4 Fig. S1** …..............................................................................................................................3

**5 Fig. S2** …..............................................................................................................................3

**6 Fig. S3** …..............................................................................................................................3

**7 Fig. S4** …..............................................................................................................................4

**8 Fig. S5** …..............................................................................................................................4

**9 Fig. S6** …..............................................................................................................................4

**10 Fig. S7** …............................................................................................................................6

**1 Table S1** GC analytical methods.

| **Substrate/products** | **Column** | **Temperature program/gradient** | **Retention time** |
| --- | --- | --- | --- |
| **1a**  **1b, 1c, 1d** | GC  CP-Chirasil-DEX CB (Agilent)  (25 m × 0.25 mm, 0.25μm)  Carrier gas: N_2_ | 100 ℃ hold 5 min  40 ℃/min to 140 ℃ hold 5 min  80 ℃/min to 180 ℃ hold 1 min | 3.18 min ethylbenzene  9.05 min (*R*)-1-phenylethanol  9.32 min (*S*)-1-phenylethanol  6.88 min acetophenone  7.42 min dodecane |
| **2a**  **2b, 2c** | GC  SH-Rtx-5  (30 m × 0.25 mm, 0.25μm)  Carrier gas: N_2_ | 80℃ hold 5 min  10 ℃/min to 150 ℃ hold 10 min  80 ℃/min to 280 ℃ hold 2 min | 4.19 min phenol  8.38 min pyrocatechol  10.11 min hydroquinone  4.48 min decane |
| **3a**  **3b** | GC  SH-Rtx-5  (30 m × 0.25 mm, 0.25μm)  Carrier gas: N_2_ | 90 ℃ hold 2 min  50 ℃/min to 150 ℃ hold 0 min  80 ℃/min to 210 ℃ hold 0 min  50 ℃/min to 280 ℃ hold 2 min | 9.37 min 4-OH β-ionone |
| **4a**  **4b, 4c** | GC  CP-Chirasil-DEX CB (Agilent)  (25 m × 0.25 mm, 0.25μm)  Carrier gas: N_2_ | 100 ℃ hold 5 min  40 ℃/min to 140 ℃ hold 5 min  80 ℃/min to 180 ℃ hold 1 min | 4.17 min styrene  7.41 min (*R*)-styrene oxide  7.57 min (*S*)-styrene oxide |
| **5a**  **5b, 5c, 5d** | GC  SH-Rtx-5  (30 m × 0.25 mm, 0.25μm)  Carrier gas: N_2_ | 80 ℃ hold 0 min  50 ℃/min to 135 ℃ hold 0 min  80 ℃/min to 240 ℃ hold 2 min | 4.38 min 2,3-isophorone epoxide  4.69 min isophorone  4.89 min 2,2,6-trimethylcyclo hexane-1,4-dione ketone  7.16 min 4-hydroxyisophoron |

**2 Table S2** Amino acid sequence of *Mfi*UPO (accession code, KAF9267131.1) from *M. fiardii* PR910.

| Site | Amino acid sequence |
| --- | --- |
| 1-60  61-120  121-180  181-240  241-260 | mknllslifi avavtastds qvdwsahrwq apgpsdtrgp cpglntlanh gflprngrni  sipmilqagh egyhiepsil tiaakvgllt spsdqpttfs ledikahgvi ehdaslsrqd  ialgdnvhfn etifstlans npgsdvyntt sagqvlqarl adslknnpnv tntdlthvir  gaesafylsv mgdpiqgvap kkfvqiffre erlpieegwk rtnisitldv vgalvdktfe  adpaweptgk ncpgiqlpdv |

**3 Table S3** The NBD activities of r*Mfi*UPO in recombinant strains constructed with pPICZ-A and pHBM905M vectors, respectively.

| Vector | NBD activity (U L^-1^) | | | |
| --- | --- | --- | --- | --- |
|  | 1-copy | 2-copy | 3-copy | 4-copy |
| pPICZ-A | 457 | 912 | 705 | 685 |
| pHBM905M | 275 | 459 | 596 | 703 |

**4 Fig. S1** Construction and confirmation of multi-copy expression cassette plasmid.


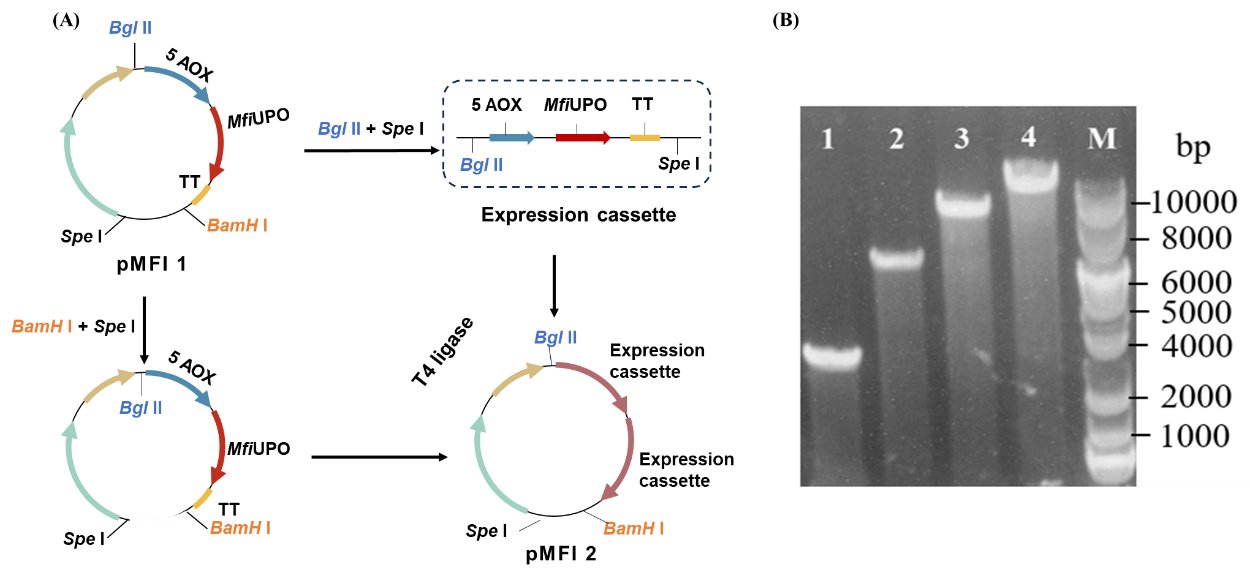


**Fig. S1 A** Construction of multi-copy expression cassette plasmid. **B** Multiple-copy recombinant plasmids confirmed by *Bgl* II digestion. 1, 2, 3 and 4 were single copy, 2-copy, 3-copy and 4-copy, respectively.

**5 Fig. S2** Activity determination of r*Mfi*UPO and deglycosylated r*Mfi*UPO.


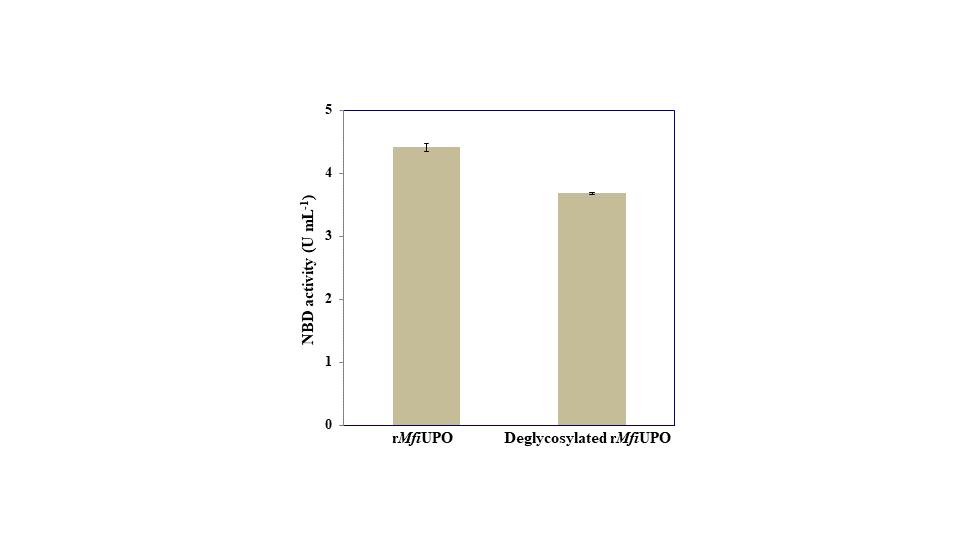


**Fig. S2** After appropriate dilution, the NBD activities of r*Mfi*UPO and deglycosylated r*Mfi*UPO were 4.41 U mL^-1^ and 3.68 U mL^-1^, respectively.

**6 Fig. S3** GC analysis of ethylbenzene and conversion products.


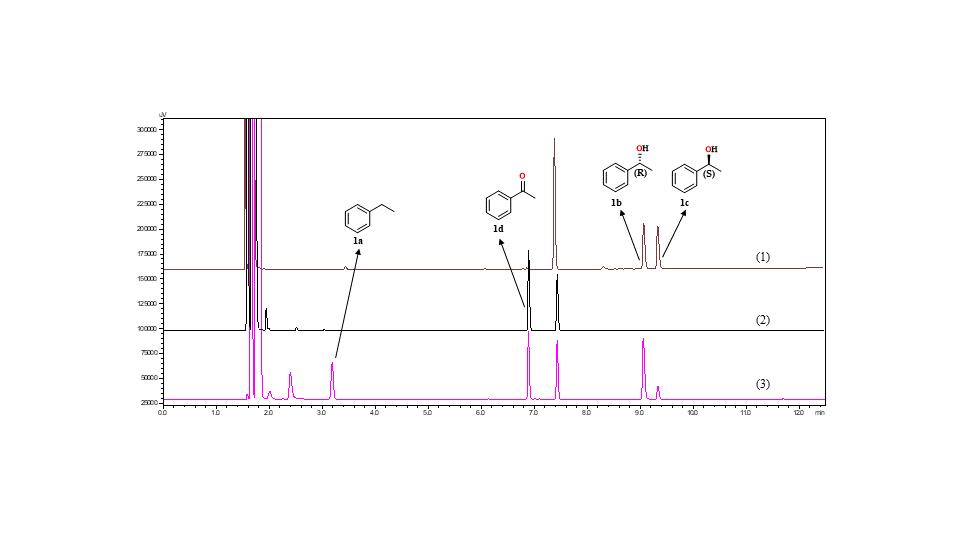


**Fig. S3** Stacked GC-FID traces of the biotransformation of ethylbenzene (1a) with r*Mfi*UPO. (1) (*R*)- and (*S*)-phenylethanol standards, (2) acetophenone standard, (3) reaction mixture.

**7 Fig. S4** GC analysis of phenol and conversion products.

**
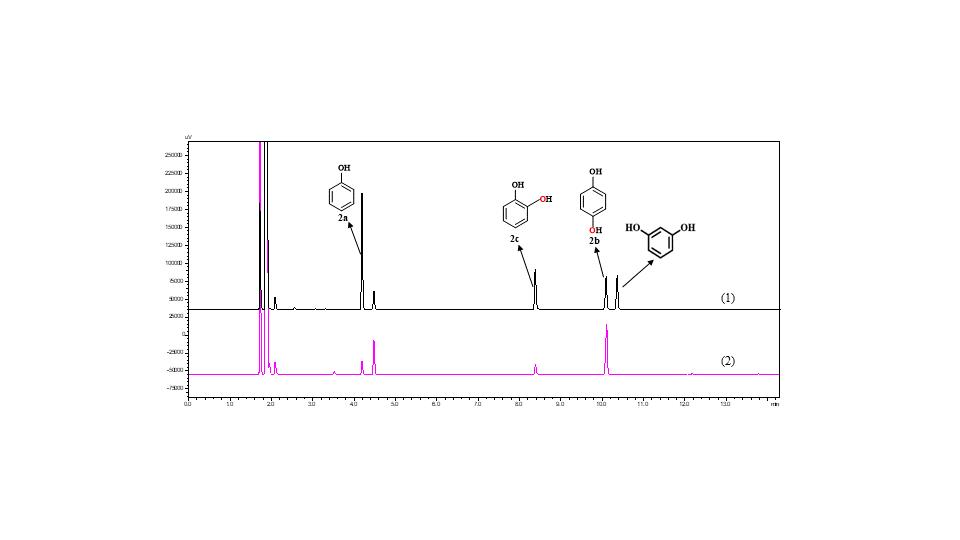
**

**Fig. S4** Stacked GC-FID traces of the biotransformation of phenol (2a) with r*Mfi*UPO. (1) phenol, catechol, hydroquinone and resorcinol standards, (2) reaction mixture.

**8 Fig. S5** GC analysis of styrene and conversion products.

**
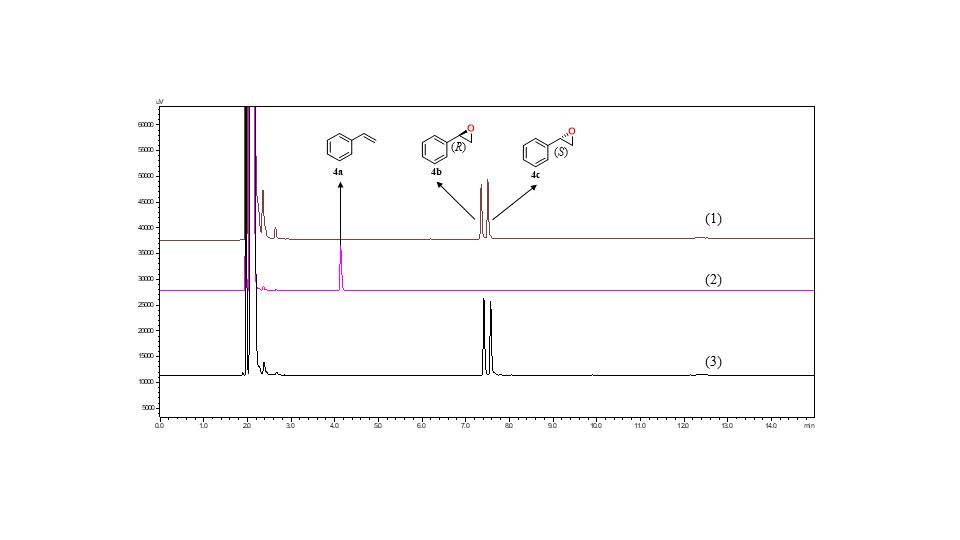
**

**Fig. S5** Stacked GC-FID traces of the biotransformation of styrene (4a) with r*Mfi*UPO. (1) (*R*)- and (*S*)-styrene oxide standards, (2) styrene standard, (3) reaction mixture.

**9 Fig. S6** GC/GC-MS analysis of isophorone and conversion products.


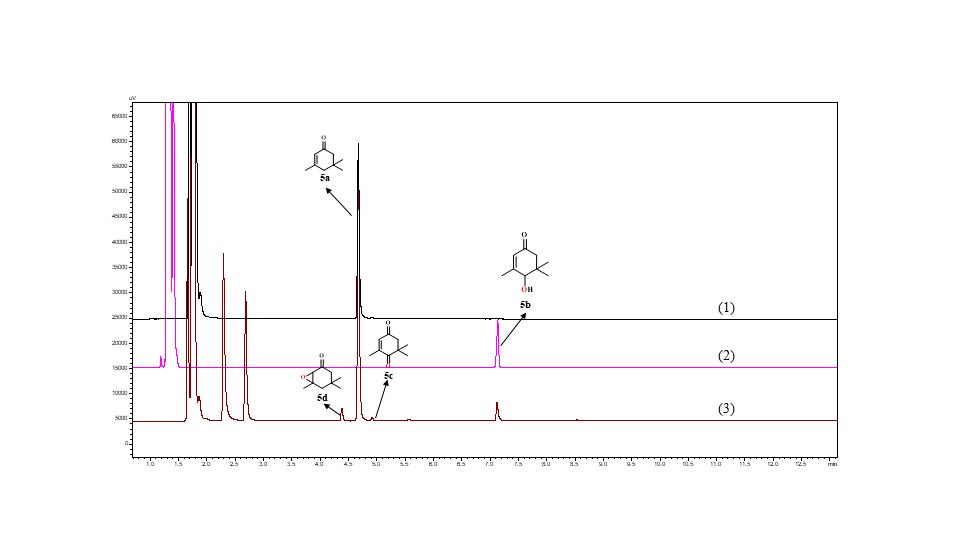


**Fig. S6a** Stacked GC-FID traces of the biotransformation of isophorone (5a) with r*Mfi*UPO. (1) Isophorone standard, (2) 4-hydroxyisophoron standard, (3) reaction mixture.


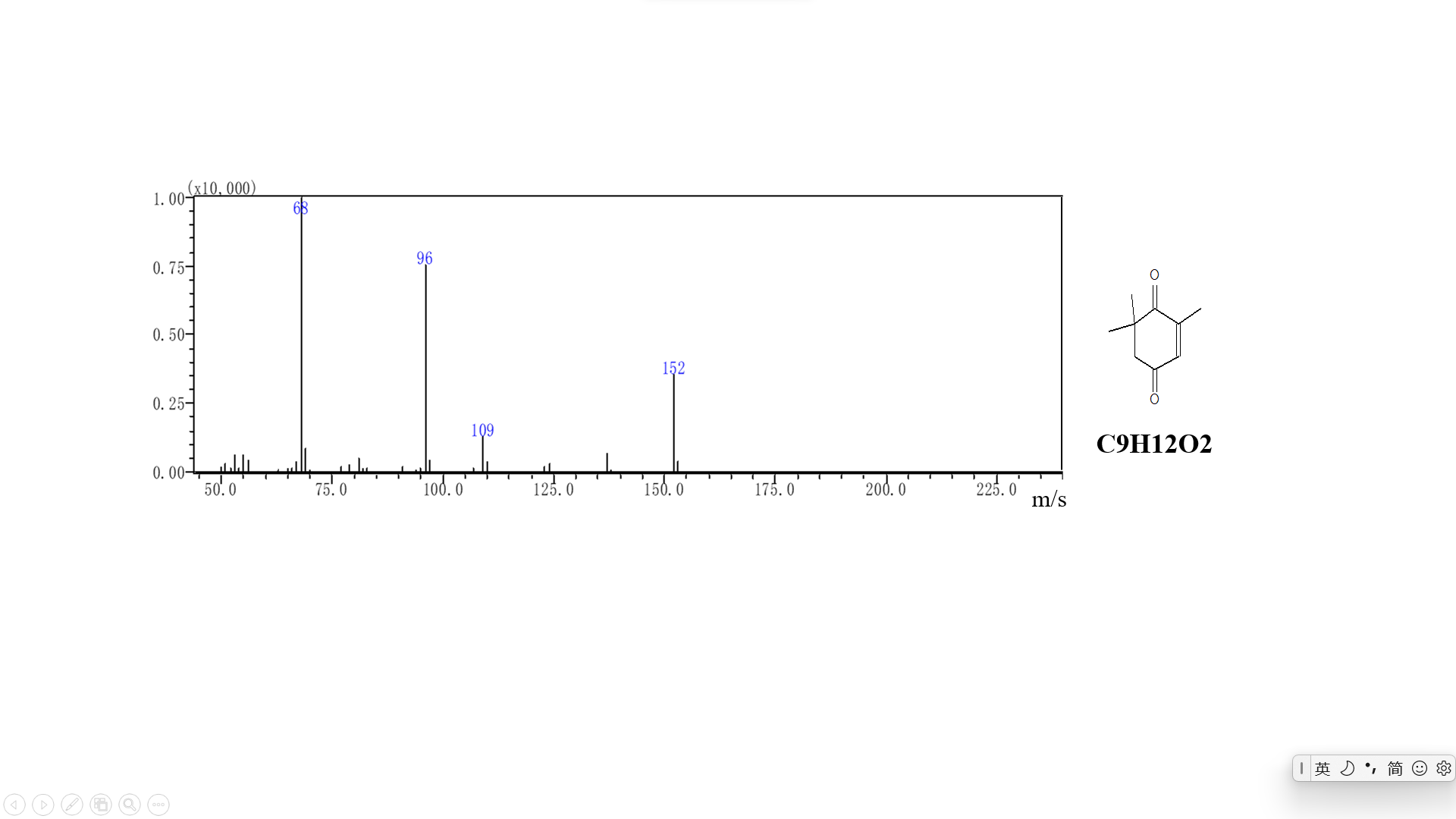


**Fig. S6b** GC-MS analysis of 2,2,6-trimethylcyclo hexane-1,4-dione ketone (5c) structural assignment. Analytical method: SH-Rtx-5 (SHIMADZU) (30 m × 0.25 mm, 0.25μm), Carrier gas: He, temperature program: 100 ℃ hold 2 min, 10 ℃/min to 190 ℃ hold 2 min, 40 ℃/min to 280 ℃ hold 3 min.


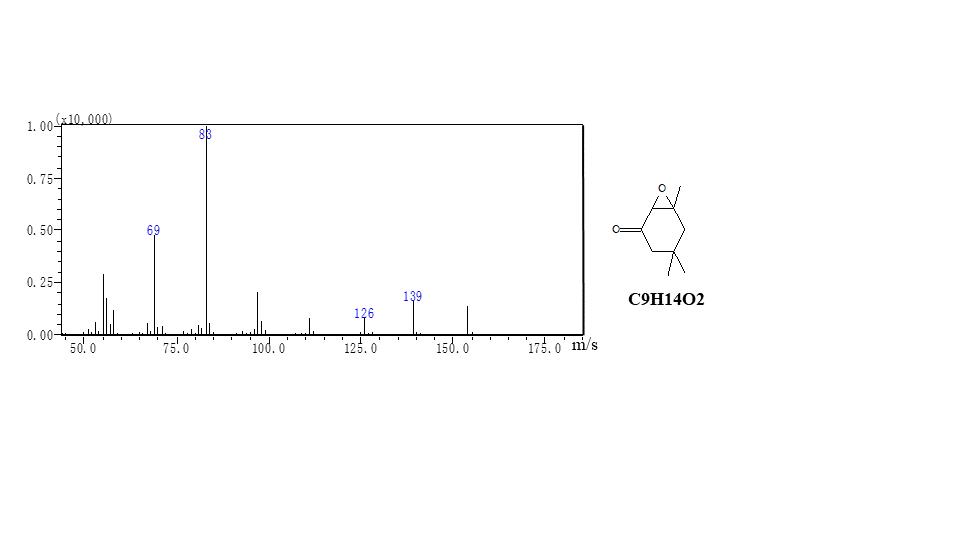


**Fig. S6c** GC-MS analysis of 2,3-isophorone epoxide (5d) structural assignment. Analytical method: SH-Rtx-5 (SHIMADZU) (30 m × 0.25 mm, 0.25μm), Carrier gas: He, temperature program: 100 ℃ hold 2 min, 10 ℃/min to 190 ℃ hold 2 min, 40 ℃/min to 280 ℃ hold 3 min.

**10 Fig. S7:** NMR spectra of 4-OH-β-ionone (**3b**), isolated from semi-preparative scale biotransformation.

**Fig. S7 Top**: ^1^H NMR (400 MHz, DMSO-*d*_6_): *δ*_H_ 7.18 (1H, d, *J* = 16.5 Hz), 6.03 (1H, d, *J* = 16.5 Hz), 4.81 (1H, d, *J* = 6.2 Hz), 2.26 (3H, s), 1.76 (3H, s), 1.75 (1H, m), 1.61 (1H, m), 1.57 (1H, m), 1.35 (1H, m), 1.03 (3H, s), 1.00 (3H, s). **Bottom:** ^13^C NMR (100 MHz, DMSO-*d*_6_): *δ*_C_ 197.9, 142.2, 137.1, 136.4, 132.3, 68.1, 34.9, 34.2, 28.5, 28.2, 27.7, 27.2, 18.3.
